# Supplementary material for: Clinical and cost-effectiveness of individualised (early) patient-directed rehabilitation versus standard rehabilitation after surgical repair of the rotator cuff of the shoulder: protocol for a multicentre, randomised controlled trial with integrated Quintet Recruitment Intervention (RaCeR 2)
Source: BMJ Open. 2024 Apr 5;14(4):e081284. doi: 10.1136/bmjopen-2023-081284 (PMC11002397; doi:10.1136/bmjopen-2023-081284)
Supplement: Supplementary data [file bmjopen-2023-081284supp001.pdf]

Derby Clinical Trials Support Unit

NHS
University Hospitals of Derby and Burton
NHS Foundation Trust

RaCeR 2 Rehabilitation following Rotator Cuff Repair

RaCeR 2 – Rehabilitation following Rotator Cuff Repair	
**CONSENT FORM**	
Local Researcher (PI):	
Participant ID:	

Please sign your initials in each box below to indicate your agreement:

- **I confirm** that I have read or have had read to me, the patient information leaflet (v2.1 05/Apr/2023) for the RaCeR 2 study, including information about the team recording our conversations. I have had the opportunity to think about the study, ask questions and have had these answered to my satisfaction. ☐

Consent for audio-recording study discussions (Quintet Recruitment Intervention – QRI)

The following section includes details of the audio recording of the conversations you have with the research team. ***Staff to tick here if this is not applicable:*** ☐

- **I consent** to the recording of discussions with me about the RaCeR 2 study and that these recordings will be written up by the study team or their authorised representatives. Yes ☐ No ☐

<u>If you say no, you can move to the next page and ignore the following two points.</u>

- **I understand** the recording of these discussions is voluntary and I am free to withdraw at any time, without giving any reason, without my medical care or legal rights being affected. ☐

- **I understand** that the recordings and write ups, may be used to support training, teaching or be shared with other researchers but I will not be identifiable from these. ☐

_______________ _______________ _______________

Name of Participant Signature Date (DD/MMM/YYYY)

Consent taken in person ☐ *Remotely* ☐ *Consent taken verbally* ☐

I am confirming that if consent is taken remotely, I will ensure a signed copy is sent to the participant.

_______________ _______________ _______________

When completed: 1 for participant; 1 for researcher site file; 1 to be kept in medical notes.

FUNDED BY NIHR | National Institute for Health and Care Research

Derby Clinical Trials Support Unit

University Hospitals of Derby and Burton NHS Foundation Trust

Name of Person seeking consent Signature Date (DD/MMM/YYYY)

Consent for RaCeR 2 study participation

If you do not wish to take part, you can ignore this page.

- **I understand** that my participation in the RaCeR 2 study is voluntary and that I am free to withdraw at any time without giving any reason, without my medical care or legal rights being affected. ☐
- **I understand** that relevant sections of my medical notes and data collected, may be looked at by authorised members of the research team from regulatory authorities or from the NHS Trust, where it is relevant to my taking part in this research. I give permission for these individuals to have access to my records, which includes identifiable information about me. ☐
- **I understand** that the information held and maintained by Derby Clinical Trials Support Unit may be used to contact me about completing the questionnaires required for the study. ☐ ☐
- **I agree** to my GP being informed of my participation in the RaCeR 2 study. Yes ☐ No ☐
- **I would like to** receive updates throughout the study, including the results ☐
- **I consent** to take part in the RaCeR 2 Study

Name of Participant	Signature	Date (DD/MMM/YYYY)

Consent taken in person ☐ *remotely* ☐
Consent taken verbally ☐

I am confirming that if consent is taken remotely, I will ensure a signed copy is sent to the participant.

When completed: 1 for participant; 1 for researcher site file; 1 to be kept in medical notes.

FUNDED BY NIHR | National Institute for Health and Care Research

Derby Clinical Trials Support Unit

University Hospitals of Derby and Burton NHS Foundation Trust

Name of Person seeking consent	Signature	Date (DD/MMM/YYYY)

When completed: 1 for participant; 1 for researcher site file; 1 to be kept in medical notes.

FUNDED BY NIHR | National Institute for Health and Care Research
