# Supplementary material for: Clinical and cost-effectiveness of individualised (early) patient-directed rehabilitation versus standard rehabilitation after surgical repair of the rotator cuff of the shoulder: protocol for a multicentre, randomised controlled trial with integrated Quintet Recruitment Intervention (RaCeR 2)
Source: BMJ Open. 2024 Apr 5;14(4):e081284. doi: 10.1136/bmjopen-2023-081284 (PMC11002397; doi:10.1136/bmjopen-2023-081284)
Supplement: Author's manuscript [file bmjopen-2023-081284.draft_revisions.pdf]

BMJ Open

BMJ Open is committed to open peer review. As part of this commitment we make the peer review history of every article we publish publicly available.

When an article is published we post the peer reviewers' comments and the authors' responses online. We also post the versions of the paper that were used during peer review. These are the versions that the peer review comments apply to.

The versions of the paper that follow are the versions that were submitted during the peer review process. They are not the versions of record or the final published versions. They should not be cited or distributed as the published version of this manuscript.

BMJ Open is an open access journal and the full, final, typeset and author-corrected version of record of the manuscript is available on our site with no access controls, subscription charges or pay-per-view fees (http://bmjopen.bmj.com).

If you have any questions on BMJ Open's open peer review process please email info.bmjopen@bmj.com

Clinical and cost-effectiveness of individualised (early) patient-directed rehabilitation versus standard rehabilitation after surgical repair of the rotator cuff of the shoulder: protocol for a multi-centre, randomised controlled trial with integrated Quintet Recruitment Intervention (RaCeR 2).

Journal:	*BMJ Open*
Manuscript ID	bmjopen-2023-081284
Article Type:	Protocol
Date Submitted by the Author:	23-Oct-2023
Complete List of Authors:	Mazuquin, Bruno ; Manchester Metropolitan University, Health Professions<br>Moffatt , Maria ; Edge Hill University Faculty of Health Social Care & Medicine<br>Realpe, Alba; University of Bristol, Population Health Sciences, Bristol Medical School; NIHR Bristol Biomedical Research Centre, University Hospitals Bristol and Weston NHS Foundation Trust and University of Bristol<br>Sherman, Rachelle; Royal Derby Hospital, Derby Clinical Trials Support Unit<br>Ireland, Katie; Royal Derby Hospital, Derby Clinical Trials Support Unit<br>Connan, Zak; Royal Derby Hospital, Derby Clinical Trials Support Unit<br>Tildsley, Jack; Royal Derby Hospital, Derby Clinical Trials Support Unit<br>Manca, Andrea; York University, Centre for Health Economics<br>Gc, Vijay; University of Huddersfield School of Human and Health Sciences<br>Foster, Nadine; The University of Queensland and Metro North Health, STARS Education and Research Alliance, Surgical Treatment and Rehabilitation Service (STARS)<br>Rees, J L; University of Oxford Nuffield Department of Orthopaedics Rheumatology and Musculoskeletal Sciences; NIHR Oxford Biomedical Research Centre<br>Drew, Steven; University Hospitals Coventry & Warwickshire NHS Trust<br>Bateman, Marcus; University Hospitals of Derby and Burton NHS Foundation Trust, Derby Shoulder Unit<br>Fakis, Apostolos; Royal Derby Hospital, Derby Clinical Trials Support Unit<br>Farnsworth, Malin; Patient and Public Involvement Representative<br>Littlewood, Chris; Edge Hill University Faculty of Health Social Care and Medicine
Keywords:	Shoulder < ORTHOPAEDIC & TRAUMA SURGERY, Physical Therapy Modalities, Randomized Controlled Trial, Orthopaedic & trauma surgery < SURGERY

Clinical and cost-effectiveness of individualised (early) patient-directed rehabilitation versus standard rehabilitation after surgical repair of the rotator cuff of the shoulder: protocol for a multi-centre, randomised controlled trial with integrated Quintet Recruitment Intervention (RaCeR 2).

Abstract

Introduction: Despite the high number of operations and surgical advancement, rehabilitation after rotator cuff repair has not progressed for over 20 years. The traditional cautious approach might be contributing to sub-optimal outcomes. Our aim is to assess whether individualised (early) patient-directed rehabilitation results in less shoulder pain and disability at 12 weeks after surgical repair of full-thickness tears of the rotator cuff compared to current standard (delayed) rehabilitation.

Methods and analysis: The rehabilitation after rotator cuff repair (RaCeR 2) study is a pragmatic multi-centre, open label, randomised controlled trial with internal pilot phase. It has a parallel group design with 1:1 allocation ratio, full health economic evaluation, and Quintet Recruitment Intervention. Adults awaiting arthroscopic surgical repair of a full-thickness tear are eligible to participate. Upon completion of surgery, 638 participants will be randomised. The intervention (individualised early patient-directed rehabilitation) includes advice to the patient to remove their sling as soon as they feel able, gradually begin using their arm as they feel able and a specific exercise programme. Sling removal and movement is progressed by the patient over time according to agreed goals and within their own pain and tolerance. The comparator (standard rehabilitation) includes advice to the patient to wear the sling for at least four weeks and only to remove while eating, washing, dressing or

For peer review only - http://bmjopen.bmj.com/site/about/guidelines.xhtml

performing specific exercises. Progression is according to specific timeframes rather than as the patient feels able. The primary outcome measure is the Shoulder Pain and Disability Index (SPADI) total score at 12-week post-randomisation. The trial timeline is 56 months in total, from September 2022.

Ethics and dissemination: We were granted ethical approval by London -Stanmore Research Ethics Committee (23/LO/0195). In addition to our trial website (www.racer2study.co.uk), we will disseminate the results via publications and presentations at national and international conferences.

Registration: ISRCTN11499185 https://doi.org/10.1186/ISRCTN11499185

Strengths and limitations of this study

- RaCeR 2 is a large randomised controlled trial investigating the clinical and cost-effectiveness of individualised early patient-directed rehabilitation after surgery to repair the torn rotator cuff of the shoulder.
- We will explore and address barriers to recruitment with the Quintet Recruitment Intervention to optimise recruitment.
- In addition to self-reported outcome measures, participants will have an ultrasound scan at 12 months to assess rotator cuff repair integrity.
- The parallel health economic analysis will assess the cost-effectiveness of individualised early patient-directed rehabilitation in comparison to standard rehabilitation.

For peer review only - http://bmjopen.bmj.com/site/about/guidelines.xhtml

Introduction

Shoulder pain associated with a rotator cuff tear can significantly affect a person's quality of life (1). The number of operations to repair rotator cuff tears has increased over time (2). In 2018/2019 direct treatment costs in the UK NHS amounted to £56.7 million (3). Following surgery, rehabilitation is provided to support patients' recovery. Current standard rehabilitation in the UK NHS, typically includes sling immobilisation for approximately one month. This has not changed for over 20 years and may be contributing to suboptimal outcomes (4).

Our systematic review of 20 randomised controlled trials (RCTs) compared the effectiveness of early versus standard post-operative rehabilitation. We found no difference between the approaches for shoulder pain and disability up to 12 months, but early rehabilitation significantly improved range of movement (5). Rotator cuff re-tear after surgery is a concern for clinicians and underpins the rationale for more cautious approaches to post-operative rehabilitation. We found no difference in repair integrity between rehabilitation approaches, but rehabilitation protocols varied and approaches described as early mobilisation were more reflective of standard rehabilitation in the UK (5).

In our RaCeR pilot, 73 patients from five NHS hospitals were randomised to individualised (early) patient-directed rehabilitation (EPDR) (advice to remove the shoulder sling as soon as able and move as symptoms allow) or standard rehabilitation (sling immobilisation for four weeks). Participants in the EPDR reported less shoulder pain and disability, returned to driving 18 days faster, had 4 fewer days lost from work over 12-weeks and fewer re-tears (30% vs 41%) (6). These findings from our RaCeR pilot, combined with our favourable assessment of feasibility and an

For peer review only - http://bmjopen.bmj.com/site/about/guidelines.xhtml

evaluation of the need for evidence using principles of value of information to research prioritisation, provided the basis for the fully powered RCT (RaCeR 2).

Objectives

Our hypothesis is that individualised EPDR is superior to standard rehabilitation for shoulder pain and disability, measured using the Shoulder Pain and Disability Index (SPADI) (7) at 12 weeks post-randomisation. The aim of RaCeR 2 is to assess the clinical and cost-effectiveness of individualised EPDR compared to NHS standard rehabilitation for pain and disability at 12 weeks after rotator cuff repair. The objectives include:

- Understanding and mitigating barriers to recruitment.
- Shoulder pain and disability at 6 and 12 months, quality of life, time to return to usual activities including work, further healthcare use, repair integrity, and adverse events to 12 months.
- Within-trial cost consequence analysis from an NHS and personal social services perspective and model-based long-term cost-effectiveness analysis.

Trial design

Pragmatic multi-centre, open label, randomised controlled trial with internal pilot. It follows a parallel group design with 1:1 allocation ratio, with full economic evaluation, and integrated Quintet Recruitment Intervention (QRI) (8).

For peer review only - http://bmjopen.bmj.com/site/about/guidelines.xhtml

Methods: participants, interventions and outcomes

This protocol paper follows the Standard Protocol Items: Recommendations for Interventional Trials (SPIRIT) (9).

Study setting

A minimum of 24 NHS orthopaedic and physiotherapy services across the UK will be opened for recruitment. The internal pilot will last six months (June to November 2023). The Trial Monitoring Group (TMG) in consultation with the Trial Steering Committee (TSC) will assess study progress and decide on progression based on the criteria in Table 1.

Table 1. Internal pilot progression criteria.

**Progression criteria**	**Red (Stop)*** **< 66%**	**Amber (Amend)**** **≥ 66% - 99%**	**Green (Go)***** **100%**
Average recruitment rate/ site/ month	< 0.7	0.7 – 1.0	1.1
Sites open	<12	12 – 17	18
Participants recruited	<50	50 – 96	97

* Red: halt, do not progress to main study.

** Amber: review areas of weakness and make amendments accordingly.

*** Green: no action required, continue to main study.

For peer review only - http://bmjopen.bmj.com/site/about/guidelines.xhtml

Eligibility criteria

Inclusion criteria:

- Adults (18 years or older) awaiting arthroscopic surgical repair of a full thickness tear of their shoulder rotator cuff, of any size.

- Able to return to the recruiting centre or affiliated site for rehabilitation supported by physiotherapists trained to deliver the study interventions.

Exclusion criteria:

- Do not have a full thickness tear at surgery and/or arthroscopic repair is not undertaken.

- Unable to provide informed consent.

- Taking part in another research study that mandates a specific post-operative rehabilitation pathway.

Recruitment and informed consent

Patients listed for rotator cuff repair surgery are screened and assessed for eligibility by trained local hospital site staff. Once an eligible patient has been identified and has been allocated a date for surgery, they are provided with an information pack about the study (including the optional QRI; more details below) and consent forms. Patients will be given the opportunity to discuss RaCeR 2 with support from an interpreter as required. We provide translated information sheets in Arabic, Bengali, Polish, Punjabi and Urdu; potential recruiting sites identified these languages as the most common languages spoken other than English in their areas. Recruiters will

For peer review only - http://bmjopen.bmj.com/site/about/guidelines.xhtml

follow up with the patient to discuss the study and answer any questions. Patients may consent to participate in neither, either or both the QRI and RaCeR 2 trial. Separate QRI clauses relating to recording of discussions about the study are included within the consent form. The process of gaining informed consent may be wholly or partly undertaken remotely or in-person depending on local site and patient circumstances. If it is not possible to get written consent, for example, if the patient is not returning to the recruiting site prior to surgery, verbal remote consent will be acceptable to avoid unnecessary burden for the patients and site staff. The consent form is completed by the recruiter indicating that consent was taken verbally, and a copy provided to the participant. This is the same for patients who consent to the audio recording of their discussion (QRI) but not to participating in RaCeR 2. Consent is fully documented within the patient's medical notes, including the method of consent (remote/ in-person and written/ verbal). Figure 1 shows the study flow diagram.

For peer review only

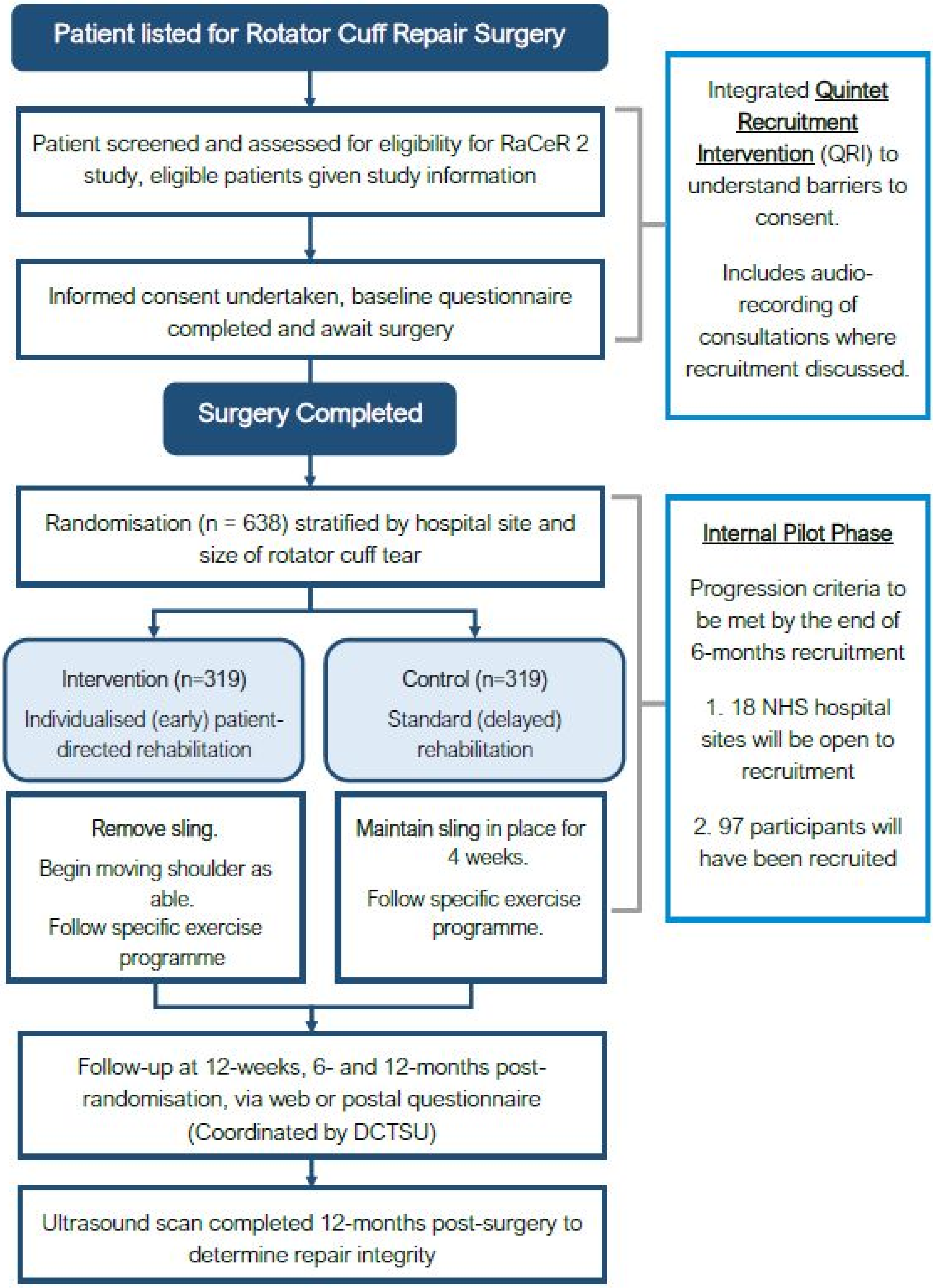

Figure 1. Study flow diagram.

For peer review only - http://bmjopen.bmj.com/site/about/guidelines.xhtml

Interventions

Individualised (early) patient-directed rehabilitation

EPDR is an individualised approach where shoulder movement, sling removal, and exercise are progressed as the participant feels able within the context of their own pain experience and tolerance. Individualised EPDR includes advice to the patient from a physiotherapist within 24-hours following surgery to remove their sling and gradually begin to actively use their arm as they feel able and within acceptable limits of pain. The advice to remove the sling is complemented by an exercise programme supervised by a physiotherapist and practised at home. After the first session with the physiotherapist, participants access follow-up with a physiotherapist according to usual care agreements. Follow-up sessions can be either face-to-face or remote.

Standard (delayed) rehabilitation

Standard (delayed) rehabilitation includes advice to the patient from a physiotherapist within 24-hours following surgery to wear their sling for four weeks except for when eating, washing, dressing, or undertaking the exercises prescribed. After the first session with the physiotherapist, participants access follow-up with a physiotherapist. The exercise programmes will be staged as follows (6):

Stage 1: Fully assisted (passive) shoulder movement.

Stage 2: Partially assisted (active assisted) with progression to full non-assisted (active) shoulder movement.

Stage 3: Resisted static exercises (isometric).

For peer review only - http://bmjopen.bmj.com/site/about/guidelines.xhtml

Stage 4: Resisted exercises through shoulder range of movement (dynamic) within limits of pain progressing to functional restoration.

Difference between current and planned care pathways

Participants in both groups agree the number of rehabilitation sessions with their physiotherapists; there is no pre-specified number of sessions. It is expected that approximately five follow-up appointments will be scheduled over the 12-week period following surgery. This means that both treatments are delivered within the parameters of current NHS physiotherapy provision. The key difference between the two rehabilitation approaches is that the individualised EPDR promotes an approach to rehabilitation which reflects patient factors including pain, pre-operative levels of function and psychological well-being. It aims to promote self-efficacy whereby the patients feel they have increased control over their recovery. Both groups will start with stage one of the specific exercise programme but the intervention group will be supported to progress through the stages as they feel able. The control group will remain at stage one for a minimum of four weeks. Patients undergoing individualised EPDR are invited to resume activities in line with their individual progress rather than pre-set timescales. Patients receiving standard rehabilitation will progress through stages based on specific time frames after surgery; stage 1 (0-4 weeks), stage 2 (4-6 weeks), stage 3 (6-8 weeks), stage 4 (8-12 weeks). Surgeons and physiotherapists will treat patients in both arms of the trial and multiple clinicians will be involved in patients' treatment in each arm.

For peer review only - http://bmjopen.bmj.com/site/about/guidelines.xhtml

Criteria for discontinuing or modifying allocated interventions

There are no specific criteria to discontinue or modify the allocated interventions. Participants can withdraw at any time. If they opt for withdrawing from the allocated treatment, they will receive standard NHS care.

Strategies to improve adherence to interventions

Participants are supported by a physiotherapist to remove their sling as they feel able or to maintain the sling in place for four weeks, depending on their allocated intervention. Participants are also supported by a physiotherapist to adhere to their prescribed exercise programme through the individual consultations and a study specific manual and website that detail the exercises and progressions. Participants are also asked to complete a sling use diary to record their time out of the sling at regular periods throughout the day for four weeks post-randomisation.

Relevant concomitant care permitted or prohibited during the trial

No concomitant care is prohibited in RaCeR 2. Other healthcare use will be collected during the trial, summarised and described.

Provisions for post-trial care

None beyond standard NHS care.

For peer review only

Outcomes

Figure 2 presents the trial schedule of outcomes and assessments. The SPADI is a validated self-report measure (7), it was more sensitive and responsive than the Oxford Shoulder Score in our RaCeR pilot and is the most used outcome measure in RCTs evaluating interventions for shoulder disorders (6). Upon receipt of informed consent, questionnaires are completed at baseline (before surgery), and at 12 weeks, 6 and 12 months after randomisation. At baseline, the questionnaire will include demographic data (e.g. date of birth, sex and ethnicity), the SPADI and EQ-5D-5L. Participants will be asked to complete a self-report sling diary for four weeks post-randomisation. Participants will complete the diary with the amount of time (hours and minutes) they were not wearing the sling at regular periods throughout the day. A self-reported questionnaire for healthcare resource use, time to return to usual activities, and any adverse events, will be completed at 12 weeks, 6 and 12 months after randomisation. At 12 months following surgery participants will undergo an ultrasound scan to evaluate repair integrity.

Participants timeline

See Figures 1 and 2.

For peer review only

Figure 2. Trial schedule of assessments and outcomes.

	Timepoint						
	**Screening**	**Baseline**	**Surgery**	**4 weeks**	**12 weeks**	**6 months**	**12 months**
**ENROLMENT**							
Eligibility	X						
Participant invitation	X						
Screening data collected	X						
Recording of recruitment appointment/ Informed consent (QRI)		X					
Randomisation			X				
**INTERVENTIONS**							
Early patient-directed rehabilitation			◆——	———	——◆		
Standard rehabilitation			◆——	———	——◆		
**ASSESSMENTS**							
Baseline questionnaire		X					
SPADI		X			X	X	X
EQ-5D-5L		X			X	X	X
Sling-use diary			◆——	——◆			
Adverse event questionnaire					X	X	X
Adverse event assessments (by clinicians)			◆——	———	———	———	——◆
Healthcare resources use					X	X	X
Ultrasound imaging							X
Assessment of treatment fidelity (by PIs)							X

PI: Principal Investigator, QRI: Quintet Recruitment Intervention, SPADI: Shoulder Pain and Disability Index, EQ-5D-5L: Euroqol five dimensions five levels.

For peer review only

Sample size

The sample size calculation was based on total SPADI score at 12 weeks, powered to detect a minimal clinically important difference of 8 points between groups (10). Assuming a standard deviation of 30 (the upper 80% confidence limit from our pilot study) (6) at 90% power and significance level 5%, and using an independent T-Test, results in 297 participants being needed per group (594 in total). However, using ANCOVA (primary analysis), adjusting for the baseline SPADI score, where correlation (r) between baseline and 12-weeks = 0.2 (data from pilot RaCeR RCT), the sample size was adjusted by (1-r2) plus one extra participant per group to 574 in total (11). In addition, adjusting for 10% non-response of SPADI questionnaire at 12 weeks, a target of 319 participants should be randomised per group, 638 in total.

Recruitment strategies

The Quintet Recruitment Intervention

We will implement the QRI aiming to optimise recruitment (8). Although our RaCeR pilot recruited 39% of those eligible, we anticipate challenges to recruitment in the main trial due to: (i) hesitance by surgeons to randomise patients (particularly older patients with larger rotator cuff tears), and (ii) challenges in participants accepting the randomised allocation due to perceived risks of individualised EPDR.

The QRI has been applied to over 25 RCTs to date, leading to insights about individual and generic recruitment issues and the development of targeted strategies to improve recruitment rates (12, 13). Rather than simply increasing the numbers of patients recruited, the QRI will aim to reduce ‘missed opportunities’ for enrolling eligible patients, while safeguarding informed consent. We will draw on insights from

For peer review only - http://bmjopen.bmj.com/site/about/guidelines.xhtml

previous application of QRI methods in RCTs, and the latest recruitment related evidence to develop materials and pre-emptive training which will support participant recruitment from the outset of RaCeR 2. Once sites open to recruitment, we will proceed to implement the QRI in two phases:

Phase 1: We will investigate recruitment issues that transpire ‘in real time’ throughout the remainder of the scheduled recruitment period. We will use mixed methods to investigate actual (rather than anticipated) issues hindering recruitment as the trial proceeds. Data collection will include:

• Semi-structured interviews with individuals involved in recruitment (‘recruiters’).

• Audio-recorded discussions between recruiters and potential participants about RaCeR 2.

• Mapping of recruitment pathways and screening log analysis.

Findings from these sources will be triangulated to generate an in-depth understanding of the ‘root-causes’ of key recruitment issues.

Phase 2: Using the results from phase 1, the QRI team will work closely with the TMG and Patient and Public Involvement (PPI) group to design and implement ‘actions’ to optimise recruitment. Actions may be applicable to all sites, specific sites, or individual recruiters, and will aim to increase the number of eligible patients approached, and/or improve conversion rates whilst safeguarding informed consent. The QRI phases will run iteratively. New avenues of enquiry will emerge throughout the conduct of the QRI, through discussion in feedback meetings and continued monitoring of screening logs.

For peer review only - http://bmjopen.bmj.com/site/about/guidelines.xhtml

We will pay close attention to screening log data before/after QRI actions to formatively evaluate the impact of actions, and the need for further investigation (Phase 1) or actions (Phase 2). Part of the QRI will entail up-front training for site staff as they open to recruitment. This training will evolve to become increasingly focused as we develop our understanding of recruitment issues, with a view to ensuring sites that open in the latter stages of the trial benefit from the QRI insights that have emerged to date.

Assignment of interventions: Allocation

Sequence generation

Upon completion of surgery, participants are randomised using minimisation. Participants are allocated on a 1:1 ratio, stratified by recruiting site and rotator cuff tear size; small (<1cm), medium (1cm to 3cm), large/massive (>3cm) or unknown.

Concealment mechanism

To ensure allocation concealment, randomisation is coordinated by Derby Clinical Trials Support Unit (DCTSU) remotely via an online randomisation system.

Implementation

The allocation sequence is generated by an online randomisation system. Following surgery, the local site team will explain to the participant their randomised allocation

For peer review only - http://bmjopen.bmj.com/site/about/guidelines.xhtml

as well as other routine post-operative requirements. An exercise manual is provided to all participants, along with the sling diary.

Assignment of interventions: Blinding (masking)

RaCeR 2 is an open label RCT. No blinding of participants, clinicians, research team or oversight committees is in place.

Data collection, management, and analysis

Plans for assessment and collection of outcomes

Following consent, the baseline questionnaire will be completed prior to surgery in-person or remotely. Completion of the baseline questionnaire will require input from local site staff and participants. The questionnaire will include the SPADI and EQ-5D-5L validated questionnaires and demographic data. The SPADI has 13 items divided into two sub-scales: pain (5 items) and disability (8 items). The responses are indicated on a visual analogue scale (0 = no pain/no difficulty and 10 = worst imaginable pain/so difficult it requires help). The items are summed and converted to a total score out of 100, a high score indicates greater pain and disability (7). The EQ-5D-5L is a generic measure of health-related quality of life. It provides a single index value for health status that can be used in a clinical or health economic evaluation (14). The EQ-5D-5L consists of questions relating to five health domains (mobility, self-care, usual activities, pain/discomfort and anxiety/depression) and respondents rate their degree of impairment using five response levels (no problems, slight, moderate, severe or extreme problems). The EQ-5D-5L is the National

For peer review only - http://bmjopen.bmj.com/site/about/guidelines.xhtml

Institute for Health and Care Excellence's (NICE) preferred measure of health-related quality of life in adults.

Follow-up questionnaires, including SPADI, EQ-5D-5L, self-report questionnaire for healthcare resource use, time to return to usual activities (including work), and any adverse events, will be completed at 12 weeks, 6 and 12 months after randomisation (+4 weeks visit window to allow for reminders). This process will be coordinated centrally by the DCTSU. Follow-up questionnaires will be available in paper or electronic format. At 12 months following surgery participants will be asked to undergo an ultrasound scan.

Plans to promote participant retention and complete follow-up

If participants do not complete their questionnaires at the expected timepoints, they will be contacted at two weeks and a minimum data collection (SPADI and adverse events) will be attempted via telephone at three weeks.

Data management

A secure electronic software platform (Dacima™) will be used to store participant study data. Each participant is assigned a participant ID for use on study forms, other study documents and the electronic database.

For peer review only - http://bmjopen.bmj.com/site/about/guidelines.xhtml

Confidentiality

All documents will be stored safely in confidential conditions in accordance with the Data Protection Act 2018 and UK General Data Protection Regulation and retained according to national legislation.

Statistical methods

Primary and secondary outcome analysis

Primary analyses will be conducted according to the intention-to-treat analysis group. ANCOVA will be used to compare total SPADI scores between individualised EPDR versus standard rehabilitation at 12 weeks after randomisation, adjusting for baseline SPADI score.

Among other secondary analyses, time to return to usual activities (work & driving) will be analysed using Kaplan-Maier curves and log rank test. Logistic regression will be undertaken to test the association between treatment groups and re-tear at 12 months. Linear regression will be used to test the association between treatment groups and time out of sling over 4 weeks. Repeated measures ANCOVA will be used to test if any treatment effect exists and has been maintained up to 12 months in terms of SPADI, and EQ-5D-5L scores. ANCOVA will be used to compare total SPADI and EQ-5D-5L scores between the treatment groups at 6 and 12 months adjusting for baseline scores. Safety analysis will be undertaken based on the per protocol analysis group. Presence of Adverse events (AEs)/Serious adverse events (SAEs) and problems after surgery will be compared between the two groups at 12 weeks, 6- and 12-months using Chi-Squared test.

For peer review only - http://bmjopen.bmj.com/site/about/guidelines.xhtml

Interim analyses

Interim descriptive analysis will be undertaken at 6 months from the start of recruitment to assess the progression criteria of the internal pilot phase. This will not include any comparison of the patient reported outcomes between the randomised groups.

Methods for additional analyses

Exploratory subgroup analysis will be undertaken for the primary endpoint at 12 weeks including an interaction term in the ANCOVA model of “rotator cuff tear size” by “treatment group”.

Definition of analysis population relating to protocol non-adherence and any statistical method to handle missing data

Per protocol analysis will consider patients with time out of sling of 222.6 hours or more over four weeks compared to those with time out of sling less than 222.6 hours base on the cut-off values from the RaCeR pilot (6). Missing values in the diary will not be included in the analysis.

Complete cases analysis will be undertaken as part of the primary endpoint analyses, where cases with missing values or those completed outside the four weeks window will be excluded in each analysis. If substantial missing data (>10% and <20%) are observed in SPADI at 12 weeks or a key prognostic covariate for the primary analysis, then multiple imputation using chained equations will be applied. Complete cases analysis will be undertaken for the secondary study outcomes.

For peer review only - http://bmjopen.bmj.com/site/about/guidelines.xhtml

Economic analysis

The perspective for both within-trial and model-based economic analyses will be that of the NHS and Personal Social Services (15). The economic analysis has three phases:

1) Development of a conceptual cost-effectiveness model structure: an initial conceptual cost-effectiveness model structure will be developed to estimate the long-term costs and quality-adjusted life year of EPDR and standard rehabilitation.

2) Within-trial cost-consequences analysis: health benefits will be quantified for changes in health-related quality of life, measured by the EQ-5D-5L. Healthcare resource use and costs observed during the trial period will be reported for each treatment group. Outcomes measured during the 12-month study period will be left undiscounted.

3) Model-based economic analysis: The long-term costs and health outcomes of EPDR and standard rehabilitation will be modelled for their impact on clinically relevant events (e.g., re-tear, re-operation), updating the state-transition model developed using the RaCeR pilot with parameters derived from data collected in RaCeR 2 and (where relevant) the published literature. Long-term predicted outcomes will be discounted at 3.5% per annum (15). The health economic analysis plan will be developed and finalised before analysis commenced and is anticipated to be disseminated in a separate publication.

For peer review only - http://bmjopen.bmj.com/site/about/guidelines.xhtml

Plans to give access to the full protocol, participant level data and statistical code

The full protocol is available at https://www.fundingawards.nihr.ac.uk/award/NIHR133874 . In the first instance, further requests for data can be made via the chief investigator (CL).

Oversight and monitoring

Composition of the coordinating centre and trial steering committee

The Chief Investigator (CL) is responsible for the conduct of the trial and will be supported by the TMG. The TMG oversees all day-to-day aspects of trial management and delivery. The independent TSC monitors the trial progress and ensures that is it is being conducted according to the protocol and the applicable regulations. The TSC has an independent chair (statistician), and four other independent members including a health economist, physiotherapist, surgeon and two PPI representatives as well as the Chief Investigator (non-independent). The TSC will meet annually. The Chief Investigator, Associate Investigator, Statistician and Trial Manager will attend the TSC meetings and report on trial progress.

Composition of the data monitoring committee, its role and reporting structure

Given the nature of RaCeR 2, a separate Data Monitoring Committee (DMC) will not be convened and the TSC will take on the data monitoring role, as agreed by the funder.

For peer review only - http://bmjopen.bmj.com/site/about/guidelines.xhtml

Adverse event reporting and harms

Number and nature of adverse events at 12-weeks, 6- and 12-months will be measured via self-report questionnaire and clinician report. Adverse events might include an increase in shoulder pain requiring additional care, e.g. prescribed medication or injection; infection up to 12-weeks post-surgery; other shoulder disorders, e.g. stiffness; rotator cuff re-rupture requiring additional care, e.g. injection, physiotherapy or surgery.

Frequency and plans for auditing trial conduct

Audits will be conducted by the Sponsor (University Hospitals of Derby & Burton NHS Foundation Trust) according to their audit plan; these may be central or site audits and may be trial or process-level audits.

Plans for communicating important protocol amendments to relevant parties

Substantial amendments will be submitted by the Sponsor to relevant regulatory bodies (Research Ethics Committee and Health Research Authority) for review and approval. The amendments will only be implemented after approval and a favourable opinion has been obtained. Non-substantial amendments will be submitted to the Health Research Authority for their approval/acknowledgment.

For peer review only - http://bmjopen.bmj.com/site/about/guidelines.xhtml

Dissemination plans

We will disseminate findings from RaCeR 2 to stakeholders via peer-reviewed publications and presentations at national and international conferences. Our website (www.racer2study.co.uk) will serve as a hub to videos describing the trial results to support patient and clinical decision making.

Patient and public involvement

PPI was embedded throughout our RaCeR pilot (6). Our PPI group informed the choice of primary outcome, directed the timing of the intervention, the reporting of ultrasound scans, and the follow-up data collection methods. They will continue to be actively involved in all stages of RaCeR 2, including development of patient-facing documents and the qualitative interview schedule for the QRI. We will work collaboratively to co-create dissemination materials such as blogs and social media posts accessible to members of the public. The co-author MF is a TMG member. Our PPI group holds regular meetings, facilitated by our PPI lead (MM).

Discussion

RaCeR 2 will be the largest RCT in the world investigating rehabilitation after rotator cuff repair (5). The findings will inform national and international clinical practice. Our primary outcome assesses pain and disability. Our comprehensive dataset will assess other outcomes of interest to the clinical community, including rotator cuff repair integrity, and the comparative cost effectiveness of individualised early patient directed rehabilitation versus standard rehabilitation.

For peer review only - http://bmjopen.bmj.com/site/about/guidelines.xhtml

Study Status

The RaCeR 2 trial (protocol version 2.2, 14th April 2023) opened to recruitment on the 1st June 2023 and is scheduled to remain open until 31st May 2025 .

For peer review only

REFERENCES

1. Carr AJ, Cooper CD, Campbell MK, Rees JL, Moser J, Beard DJ, et al. Clinical effectiveness and cost-effectiveness of open and arthroscopic rotator cuff repair the UK Rotator Cuff Surgery (UKUFF) randomised trial . Health technology assessment (Winchester, England). 2015;19(80):1-218.
2. Karjalainen TV, Jain NB, Heikkinen J, Johnston RV, Page CM, Buchbinder R. Surgery for rotator cuff tears. The Cochrane database of systematic reviews. 2019;12(12):Cd013502.
3. HES. Hospital Episode Statistics. Hospital Admitted Patient Care Activity 2018-19 [Internet]. 2019 [cited 2020 May 18].
4. Littlewood C, Mazuquin B, Moffatt M, Bateman M. Rehabilitation following rotator cuff repair: A survey of current practice (2020). Musculoskeletal Care. 2021;19(2):165-71.
5. Mazuquin B, Moffatt M, Gill P, Selfe J, Rees J, Drew S, et al. Effectiveness of early versus delayed rehabilitation following rotator cuff repair: Systematic review and meta-analyses. PLOS ONE. 2021;16(5):e0252137.
6. Littlewood C, Bateman M, Butler-Walley S, Bathers S, Bromley K, Lewis M, et al. Rehabilitation following rotator cuff repair: A multi-centre pilot & feasibility randomised controlled trial (RaCeR). Clinical Rehabilitation. 2020:0269215520978859.
7. MacDermid JC, Solomon P, Prkachin K. The Shoulder Pain and Disability Index demonstrates factor, construct and longitudinal validity. BMC Musculoskelet Disord. 2006;7:12.
8. Donovan JL, Rooshenas L, Jepson M, Elliott D, Wade J, Avery K, et al. Optimising recruitment and informed consent in randomised controlled trials: the development and implementation of the Quintet Recruitment Intervention (QRI). Trials. 2016;17(1):283.
9. Chan AW, Tetzlaff JM, Altman DG, Laupacis A, Gøtzsche PC, Krleža-Jerić K, et al. SPIRIT 2013 statement: defining standard protocol items for clinical trials. Ann Intern Med. 2013;158(3):200-7.
10. Buchbinder R, Ramiro S, Huang H, Gagnier JJ, Jia Y, Whittle SL. Measures of Adult Shoulder Function. Arthritis Care & Research. 2020;72(S10):250-93.
11. Borm GF, Fransen J, Lemmens WA. A simple sample size formula for analysis of covariance in randomized clinical trials. J Clin Epidemiol. 2007;60(12):1234-8.
12. Rooshenas L, Scott LJ, Blazeby JM, Rogers CA, Tilling KM, Husbands S, et al. The QuinteT Recruitment Intervention supported five randomized trials to recruit to target: a mixed-methods evaluation. Journal of Clinical Epidemiology. 2019;106:108-20.
13. Rooshenas L, Elliott D, Wade J, Jepson M, Paramasivan S, Strong S, et al. Conveying Equipoise during Recruitment for Clinical Trials: Qualitative Synthesis of Clinicians' Practices across Six Randomised Controlled Trials. PLOS Medicine. 2016;13(10):e1002147.
14. Hernández Alava M, Wailoo AJ, Ara R. Tails from the peak district: adjusted limited dependent variable mixture models of EQ-5D questionnaire health state utility values. Value in health : the journal of the International Society for Pharmacoeconomics and Outcomes Research. 2012;15(3):550-61.

For peer review only - http://bmjopen.bmj.com/site/about/guidelines.xhtml

15. National Institute for Health and Clinical Excellence. Guide to the methods of technology appraisal, 2013.

For peer review only

For peer review only - http://bmjopen.bmj.com/site/about/guidelines.xhtml

Acknowledgements: NA

Authors' contributions: CL, MB, NF, BM, MM, AR, AM, VG, JR, ST and AF conceived of the study and were involved, alongside RS, KI and MF in developing the design and protocol. CL, BM, MM, AM, VG, AR, NF, JR, SD, MB and AF secured funding for the study. BM drafted the manuscript and all other authors reviewed and provided feedback on drafts. All authors read and approved the final version of the manuscript.

Funding statement: This trial is funded by the NIHR HTA (Ref: 133874). The views expressed are those of the author(s) and not necessarily those of the NIHR, the Department of Health and Social Care, or the NHS.

Availability of data and materials: Data produced from the trial will be made available to other researchers upon request, subject to approval by the Sponsor.

Ethics approval and consent to participate: Ethical approval was granted by London - Stanmore Research Ethics Committee (23/LO/0195). All participants must provide consent before participating, after receiving a full written and verbal explanation of the study's aims, procedures and risks.

Consent for publication: Consent for publication is not applicable as there are no identifying images or other personal details of participants presented.

Competing interests:

SD holds education consultancy contracts with Stryker, Smith and Nephew and Arthrex for teaching and training.

Word count: 3999 words

For peer review only - http://bmjopen.bmj.com/site/about/guidelines.xhtml

STANDARD PROTOCOL ITEMS: RECOMMENDATIONS FOR INTERVENTIONAL TRIALS

SPIRIT 2013 Checklist: Recommended items to address in a clinical trial protocol and related documents*

Section/item	ItemNo	Description	Page
**Administrative information**			
Title	1	Descriptive title identifying the study design, population, interventions, and, if applicable, trial acronym	1
Trial registration	2a	Trial identifier and registry name. If not yet registered, name of intended registry	2
	2b	All items from the World Health Organization Trial Registration Data Set	NA
Protocol version	3	Date and version identifier	25
Funding	4	Sources and types of financial, material, and other support	28
Roles and responsibilities	5a	Names, affiliations, and roles of protocol contributors	1 and 28
	5b	Name and contact information for the trial sponsor	23
	5c	Role of study sponsor and funders, if any, in study design; collection, management, analysis, and interpretation of data; writing of the report; and the decision to submit the report for publication, including whether they will have ultimate authority over any of these activities	28
	5d	Composition, roles, and responsibilities of the coordinating centre, steering committee, endpoint adjudication committee, data management team, and other individuals or groups overseeing the trial, if applicable (see Item 21a for data monitoring committee)	22
**Introduction**			
Background and rationale	6a	Description of research question and justification for undertaking the trial, including summary of relevant studies (published and unpublished) examining benefits and harms for each intervention	3-4
	6b	Explanation for choice of comparators	9-10

For peer review only

Objectives	7	Specific objectives or hypotheses	4
Trial design	8	Description of trial design including type of trial (eg, parallel group, crossover, factorial, single group), allocation ratio, and framework (eg, superiority, equivalence, noninferiority, exploratory)	4
**Methods: Participants, interventions, and outcomes**			
Study setting	9	Description of study settings (eg, community clinic, academic hospital) and list of countries where data will be collected. Reference to where list of study sites can be obtained	5
Eligibility criteria	10	Inclusion and exclusion criteria for participants. If applicable, eligibility criteria for study centres and individuals who will perform the interventions (eg, surgeons, psychotherapists)	6
Interventions	11a	Interventions for each group with sufficient detail to allow replication, including how and when they will be administered	9
	11b	Criteria for discontinuing or modifying allocated interventions for a given trial participant (eg, drug dose change in response to harms, participant request, or improving/worsening disease)	11
	11c	Strategies to improve adherence to intervention protocols, and any procedures for monitoring adherence (eg, drug tablet return, laboratory tests)	11
	11d	Relevant concomitant care and interventions that are permitted or prohibited during the trial	11
Outcomes	12	Primary, secondary, and other outcomes, including the specific measurement variable (eg, systolic blood pressure), analysis metric (eg, change from baseline, final value, time to event), method of aggregation (eg, median, proportion), and time point for each outcome. Explanation of the clinical relevance of chosen efficacy and harm outcomes is strongly recommended	12
Participant timeline	13	Time schedule of enrolment, interventions (including any run-ins and washouts), assessments, and visits for participants. A schematic diagram is highly recommended (see Figure)	12-13 (Figure 2)

For peer review only - http://bmjopen.bmj.com/site/about/guidelines.xhtml

Sample size	14	Estimated number of participants needed to achieve study objectives and how it was determined, including clinical and statistical assumptions supporting any sample size calculations	14
Recruitment	15	Strategies for achieving adequate participant enrolment to reach target sample size	14-16
**Methods: Assignment of interventions (for controlled trials)**			
Allocation:			
Sequence generation	16a	Method of generating the allocation sequence (eg, computer-generated random numbers), and list of any factors for stratification. To reduce predictability of a random sequence, details of any planned restriction (eg, blocking) should be provided in a separate document that is unavailable to those who enrol participants or assign interventions	16
Allocation concealment mechanism	16b	Mechanism of implementing the allocation sequence (eg, central telephone; sequentially numbered, opaque, sealed envelopes), describing any steps to conceal the sequence until interventions are assigned	16
Implementation	16c	Who will generate the allocation sequence, who will enrol participants, and who will assign participants to interventions	16
Blinding (masking)	17a	Who will be blinded after assignment to interventions (eg, trial participants, care providers, outcome assessors, data analysts), and how	17
	17b	If blinded, circumstances under which unblinding is permissible, and procedure for revealing a participant's allocated intervention during the trial	NA
**Methods: Data collection, management, and analysis**			
Data collection methods	18a	Plans for assessment and collection of outcome, baseline, and other trial data, including any related processes to promote data quality (eg, duplicate measurements, training of assessors) and a description of study instruments (eg, questionnaires, laboratory tests) along with their reliability and validity, if known. Reference to where data collection forms can be found, if not in the protocol	17
	18b	Plans to promote participant retention and complete follow-up, including list of any outcome data to be collected for participants who discontinue or deviate from intervention protocols	18

For peer review only - http://bmjopen.bmj.com/site/about/guidelines.xhtml

Data management	19	Plans for data entry, coding, security, and storage, including any related processes to promote data quality (eg, double data entry; range checks for data values). Reference to where details of data management procedures can be found, if not in the protocol	18
Statistical methods	20a	Statistical methods for analysing primary and secondary outcomes. Reference to where other details of the statistical analysis plan can be found, if not in the protocol	19
	20b	Methods for any additional analyses (eg, subgroup and adjusted analyses)	20
	20c	Definition of analysis population relating to protocol non-adherence (eg, as randomised analysis), and any statistical methods to handle missing data (eg, multiple imputation)	20
**Methods: Monitoring**			
Data monitoring	21a	Composition of data monitoring committee (DMC); summary of its role and reporting structure; statement of whether it is independent from the sponsor and competing interests; and reference to where further details about its charter can be found, if not in the protocol. Alternatively, an explanation of why a DMC is not needed	22
	21b	Description of any interim analyses and stopping guidelines, including who will have access to these interim results and make the final decision to terminate the trial	20
Harms	22	Plans for collecting, assessing, reporting, and managing solicited and spontaneously reported adverse events and other unintended effects of trial interventions or trial conduct	23
Auditing	23	Frequency and procedures for auditing trial conduct, if any, and whether the process will be independent from investigators and the sponsor	23
**Ethics and dissemination**			
Research ethics approval	24	Plans for seeking research ethics committee/institutional review board (REC/IRB) approval	28

For peer review only - http://bmjopen.bmj.com/site/about/guidelines.xhtml

Protocol amendments	25	Plans for communicating important protocol modifications (eg, changes to eligibility criteria, outcomes, analyses) to relevant parties (eg, investigators, REC/IRBs, trial participants, trial registries, journals, regulators)	23
Consent or assent	26a	Who will obtain informed consent or assent from potential trial participants or authorised surrogates, and how (see Item 32)	6
	26b	Additional consent provisions for collection and use of participant data and biological specimens in ancillary studies, if applicable	NA
Confidentiality	27	How personal information about potential and enrolled participants will be collected, shared, and maintained in order to protect confidentiality before, during, and after the trial	18-19
Declaration of interests	28	Financial and other competing interests for principal investigators for the overall trial and each study site	28
Access to data	29	Statement of who will have access to the final trial dataset, and disclosure of contractual agreements that limit such access for investigators	22
Ancillary and post-trial care	30	Provisions, if any, for ancillary and post-trial care, and for compensation to those who suffer harm from trial participation	11
Dissemination policy	31a	Plans for investigators and sponsor to communicate trial results to participants, healthcare professionals, the public, and other relevant groups (eg, via publication, reporting in results databases, or other data sharing arrangements), including any publication restrictions	24
	31b	Authorship eligibility guidelines and any intended use of professional writers	28
	31c	Plans, if any, for granting public access to the full protocol, participant-level dataset, and statistical code	22
**Appendices**			
Informed consent materials	32	Model consent form and other related documentation given to participants and authorised surrogates	Not available
Biological specimens	33	Plans for collection, laboratory evaluation, and storage of biological specimens for genetic or molecular analysis in the current trial and for future use in ancillary studies, if applicable	NA

For peer review only - http://bmjopen.bmj.com/site/about/guidelines.xhtml

*It is strongly recommended that this checklist be read in conjunction with the SPIRIT 2013 Explanation & Elaboration for important clarification on the items. Amendments to the protocol should be tracked and dated. The SPIRIT checklist is copyrighted by the SPIRIT Group under the Creative Commons "Attribution-NonCommercial-NoDerivs 3.0 Unported" license.

For peer review only

BMJ Open

Clinical and cost-effectiveness of individualised (early) patient-directed rehabilitation versus standard rehabilitation after surgical repair of the rotator cuff of the shoulder: protocol for a multi-centre, randomised controlled trial with integrated Quintet Recruitment Intervention (RaCeR 2).

Journal:	*BMJ Open*
Manuscript ID	bmjopen-2023-081284.R1
Article Type:	Protocol
Date Submitted by the Author:	05-Feb-2024

Complete List of Authors:	Mazuquin, Bruno ; Manchester Metropolitan University, Health Professions<br>Moffatt , Maria ; Edge Hill University Faculty of Health Social Care & Medicine<br>Realpe, Alba; University of Bristol, Population Health Sciences, Bristol Medical School; NIHR Bristol Biomedical Research Centre, University Hospitals Bristol and Weston NHS Foundation Trust and University of Bristol<br>Sherman, Rachelle; Royal Derby Hospital, Derby Clinical Trials Support Unit<br>Ireland, Katie; Royal Derby Hospital, Derby Clinical Trials Support Unit<br>Connan, Zak; Royal Derby Hospital, Derby Clinical Trials Support Unit<br>Tildsley, Jack; Royal Derby Hospital, Derby Clinical Trials Support Unit<br>Manca, Andrea; York University, Centre for Health Economics<br>Gc, Vijay; University of Huddersfield School of Human and Health Sciences<br>Foster, Nadine; The University of Queensland and Metro North Health, STARS Education and Research Alliance, Surgical Treatment and Rehabilitation Service (STARS)<br>Rees, J L; University of Oxford Nuffield Department of Orthopaedics Rheumatology and Musculoskeletal Sciences; NIHR Oxford Biomedical Research Centre<br>Drew, Steven; University Hospitals Coventry & Warwickshire NHS Trust<br>Bateman, Marcus; University Hospitals of Derby and Burton NHS Foundation Trust, Derby Shoulder Unit<br>Fakis, Apostolos; Royal Derby Hospital, Derby Clinical Trials Support Unit<br>Farnsworth, Malin; Patient and Public Involvement Representative<br>Littlewood, Chris; Edge Hill University Faculty of Health Social Care and Medicine

**Primary Subject Heading**:	Rehabilitation medicine
Secondary Subject Heading:	Rehabilitation medicine
Keywords:	Shoulder < ORTHOPAEDIC & TRAUMA SURGERY, Physical Therapy

	Modalities, Randomized Controlled Trial, Orthopaedic & trauma surgery < SURGERY

SCHOLARONE™
Manuscripts

Clinical and cost-effectiveness of individualised (early) patient-directed rehabilitation versus standard rehabilitation after surgical repair of the rotator cuff of the shoulder: protocol for a multi-centre, randomised controlled trial with integrated Quintet Recruitment Intervention (RaCeR 2).

Abstract

Introduction: Despite the high number of operations and surgical advancement, rehabilitation after rotator cuff repair has not progressed for over 20 years. The traditional cautious approach might be contributing to sub-optimal outcomes. Our aim is to assess whether individualised (early) patient-directed rehabilitation results in less shoulder pain and disability at 12 weeks after surgical repair of full-thickness tears of the rotator cuff compared to current standard (delayed) rehabilitation.

Methods and analysis: The rehabilitation after rotator cuff repair (RaCeR 2) study is a pragmatic multi-centre, open label, randomised controlled trial with internal pilot phase. It has a parallel group design with 1:1 allocation ratio, full health economic evaluation, and Quintet Recruitment Intervention. Adults awaiting arthroscopic surgical repair of a full-thickness tear are eligible to participate. Upon completion of surgery, 638 participants will be randomised. The intervention (individualised early patient-directed rehabilitation) includes advice to the patient to remove their sling as soon as they feel able, gradually begin using their arm as they feel able and a specific exercise programme. Sling removal and movement is progressed by the patient over time according to agreed goals and within their own pain and tolerance. The comparator (standard rehabilitation) includes advice to the patient to wear the sling for at least four weeks and only to remove while eating, washing, dressing or

For peer review only - http://bmjopen.bmj.com/site/about/guidelines.xhtml

performing specific exercises. Progression is according to specific timeframes rather than as the patient feels able. The primary outcome measure is the Shoulder Pain and Disability Index (SPADI) total score at 12-week post-randomisation. The trial timeline is 56 months in total, from September 2022.

Registration: ISRCTN11499185 https://doi.org/10.1186/ISRCTN11499185

Strengths and limitations of this study

- RaCeR 2 is a large randomised controlled trial investigating the clinical and cost-effectiveness of individualised early patient-directed rehabilitation after surgery to repair the torn rotator cuff of the shoulder.
- We will explore and address barriers to recruitment with the Quintet Recruitment Intervention to optimise recruitment.
- In addition to self-reported outcome measures, participants will have an ultrasound scan at 12 months to assess rotator cuff repair integrity.
- The parallel health economic analysis will assess the cost-effectiveness of individualised early patient-directed rehabilitation in comparison to standard rehabilitation.

For peer review only

Introduction

Shoulder pain associated with a rotator cuff tear can significantly affect a person's quality of life (1). The number of operations to repair rotator cuff tears has increased over time (2). In 2018/2019 direct treatment costs in the UK NHS amounted to £56.7 million (3). Following surgery, rehabilitation is provided to support patients' recovery. Current standard rehabilitation in the UK NHS, typically includes sling immobilisation for approximately one month. This has not changed for over 20 years and may be contributing to suboptimal outcomes (4).

Our systematic review of 20 randomised controlled trials (RCTs) compared the effectiveness of early versus standard post-operative rehabilitation. We found no difference between the approaches for shoulder pain and disability up to 12 months, but early rehabilitation significantly improved range of movement (5). Rotator cuff re-tear after surgery is a concern for clinicians and underpins the rationale for more cautious approaches to post-operative rehabilitation. We found no difference in repair integrity between rehabilitation approaches, but rehabilitation protocols varied and approaches described as early mobilisation were more reflective of standard rehabilitation in the UK (5).

In our RaCeR pilot, 73 patients from five NHS hospitals were randomised to individualised (early) patient-directed rehabilitation (EPDR) (advice to remove the shoulder sling as soon as able and move as symptoms allow) or standard rehabilitation (sling immobilisation for four weeks). Participants in the EPDR reported less shoulder pain and disability, returned to driving 18 days faster, had 4 fewer days lost from work over 12-weeks and fewer re-tears (30% vs 41%) (6). These findings from our RaCeR pilot, combined with our favourable assessment of feasibility and an

For peer review only

evaluation of the need for evidence using principles of value of information to research prioritisation, provided the basis for the fully powered RCT (RaCeR 2).

Objectives

Our hypothesis is that individualised EPDR is superior to standard rehabilitation for shoulder pain and disability, measured using the Shoulder Pain and Disability Index (SPADI) (7) at 12 weeks post-randomisation. The aim of RaCeR 2 is to assess the clinical and cost-effectiveness of individualised EPDR compared to NHS standard rehabilitation for pain and disability at 12 weeks after rotator cuff repair. The objectives include:

- Understanding and mitigating barriers to recruitment.
- Shoulder pain and disability at 6 and 12 months, quality of life, time to return to drive and usual activities including work, further healthcare use, repair integrity, and adverse events to 12 months.
- Within-trial cost consequence analysis from an NHS and personal social services perspective and model-based long-term cost-effectiveness analysis.

Trial design

Pragmatic multi-centre, open label, randomised controlled trial with internal pilot. It follows a parallel group design with 1:1 allocation ratio, with full economic evaluation, and integrated Quintet Recruitment Intervention (QRI) (8).

For peer review only - http://bmjopen.bmj.com/site/about/guidelines.xhtml

Methods: participants, interventions and outcomes

This protocol paper follows the Standard Protocol Items: Recommendations for Interventional Trials (SPIRIT) (9).

Study setting

A minimum of 24 NHS orthopaedic and physiotherapy services across the UK will be opened for recruitment. The internal pilot will last six months (June to November 2023). The Trial Monitoring Group (TMG) in consultation with the Trial Steering Committee (TSC) will assess study progress and decide on progression based on the criteria in Table 1.

Table 1. Internal pilot progression criteria.

**Progression criteria**	**Red (Stop)*** **< 66%**	**Amber (Amend)**** **≥ 66% - 99%**	**Green (Go)***** **100%**
Average recruitment rate/ site/ month	< 0.7	0.7 – 1.0	1.1
Sites open	<12	12 – 17	18
Participants recruited	<50	50 – 96	97

* Red: halt, do not progress to main study.

** Amber: review areas of weakness and make amendments accordingly.

*** Green: no action required, continue to main study.

For peer review only - http://bmjopen.bmj.com/site/about/guidelines.xhtml

Eligibility criteria

Inclusion criteria:

- Adults (18 years or older) awaiting arthroscopic surgical repair of a full thickness tear of their shoulder rotator cuff, of any size.

- Able to return to the recruiting centre or affiliated site for rehabilitation supported by physiotherapists trained to deliver the study interventions.

Exclusion criteria:

- Do not have a full thickness tear at surgery and/or arthroscopic repair is not undertaken.

- Unable to provide informed consent.

- Taking part in another research study that mandates a specific post-operative rehabilitation pathway.

Recruitment and informed consent

Patients listed for rotator cuff repair surgery are screened and assessed for eligibility by trained local hospital site staff. Once an eligible patient has been identified and has been allocated a date for surgery, they are provided with an information pack about the study (including the optional QRI; more details below) and consent forms. Patients will be given the opportunity to discuss RaCeR 2 with support from an interpreter as required. We provide translated information sheets in Arabic, Bengali, Polish, Punjabi and Urdu; potential recruiting sites identified these languages as the most common languages spoken other than English in their areas. Recruiters will

For peer review only - http://bmjopen.bmj.com/site/about/guidelines.xhtml

follow up with the patient to discuss the study and answer any questions. Patients may consent to participate in neither, either or both the QRI and RaCeR 2 trial. Separate QRI clauses relating to recording of discussions about the study are included within the consent form. The process of gaining informed consent may be wholly or partly undertaken remotely or in-person depending on local site and patient circumstances. If it is not possible to get written consent, for example, if the patient is not returning to the recruiting site prior to surgery, verbal remote consent will be acceptable to avoid unnecessary burden for the patients and site staff. The consent form is completed by the recruiter indicating that consent was taken verbally, and a copy provided to the participant. This is the same for patients who consent to the audio recording of their discussion (QRI) but not to participating in RaCeR 2. Consent is fully documented within the patient's medical notes, including the method of consent (remote/ in-person and written/ verbal). Figure 1 shows the study flow diagram.

Please insert Figure 1 here.

Figure 1. Study flow diagram.

Interventions

Individualised (early) patient-directed rehabilitation

EPDR is an individualised approach where shoulder movement, sling removal, and exercise are progressed as the participant feels able within the context of their own pain experience and tolerance. Individualised EPDR includes advice to the patient

For peer review only - http://bmjopen.bmj.com/site/about/guidelines.xhtml

from a physiotherapist within 24-hours following surgery to remove their sling and gradually begin to actively use their arm as they feel able and within acceptable limits of pain. The advice to remove the sling is complemented by an exercise programme supervised by a physiotherapist and practised at home. After the first session with the physiotherapist, participants access follow-up with a physiotherapist according to usual care agreements. Follow-up sessions can be either face-to-face or remote.

Standard (delayed) rehabilitation

Standard (delayed) rehabilitation includes advice to the patient from a physiotherapist within 24-hours following surgery to wear their sling for four weeks except for when eating, washing, dressing, or undertaking the exercises prescribed. After the first session with the physiotherapist, participants access follow-up with a physiotherapist. The exercise programmes will be staged as follows (6):

Stage 1: Fully assisted (passive) shoulder movement.

Stage 2: Partially assisted (active assisted) with progression to full non-assisted (active) shoulder movement.

Stage 3: Resisted static exercises (isometric).

Stage 4: Resisted exercises through shoulder range of movement (dynamic) within limits of pain progressing to functional restoration.

For peer review only - http://bmjopen.bmj.com/site/about/guidelines.xhtml

Difference between current and planned care pathways

Participants in both groups agree the number of rehabilitation sessions with their physiotherapists; there is no pre-specified number of sessions. It is expected that approximately five follow-up appointments will be scheduled over the 12-week period following surgery. This means that both treatments are delivered within the parameters of current NHS physiotherapy provision. The key difference between the two rehabilitation approaches is that the individualised EPDR promotes an approach to rehabilitation which reflects patient factors including pain, pre-operative levels of function and psychological well-being. It aims to promote self-efficacy whereby the patients feel they have increased control over their recovery. Both groups will start with stage one of the specific exercise programme but the intervention group will be supported to progress through the stages as they feel able. The control group will remain at stage one for a minimum of four weeks. Patients undergoing individualised EPDR are invited to resume activities in line with their individual progress rather than pre-set timescales. Patients receiving standard rehabilitation will progress through stages based on specific time frames after surgery; stage 1 (0-4 weeks), stage 2 (4-6 weeks), stage 3 (6-8 weeks), stage 4 (8-12 weeks). Surgeons and physiotherapists will treat patients in both arms of the trial and multiple clinicians will be involved in patients' treatment in each arm.

Criteria for discontinuing or modifying allocated interventions

There are no specific criteria to discontinue or modify the allocated interventions. Participants can withdraw at any time. If they opt for withdrawing from the allocated treatment, they will receive standard NHS care.

For peer review only - http://bmjopen.bmj.com/site/about/guidelines.xhtml

Strategies to improve adherence to interventions

Participants are supported by a physiotherapist to remove their sling as they feel able or to maintain the sling in place for four weeks, depending on their allocated intervention. Participants are also supported by a physiotherapist to adhere to their prescribed exercise programme through the individual consultations and a study specific manual and website that detail the exercises and progressions. Participants are also asked to complete a sling use diary to record their time out of the sling at regular periods throughout the day for four weeks post-randomisation.

Relevant concomitant care permitted or prohibited during the trial

No concomitant care is prohibited in RaCeR 2. Other healthcare use will be collected during the trial, summarised and described.

Provisions for post-trial care

None beyond standard NHS care.

Outcomes

Figure 2 presents the trial schedule of outcomes and assessments. Upon receipt of informed consent (Supplemental material 1), questionnaires are completed at baseline (before surgery), and at 12 weeks, 6 and 12 months after randomisation. At

For peer review only - http://bmjopen.bmj.com/site/about/guidelines.xhtml

baseline, the questionnaire will include demographic data (e.g. date of birth, sex and ethnicity), the SPADI and EQ-5D-5L.

Primary outcome measure

Shoulder pain and disability at 12-weeks post-randomisation will be measured using the SPADI. The SPADI is a validated self-report measure (7), it was more sensitive and responsive than the Oxford Shoulder Score in our RaCeR pilot and is the most used outcome measure in RCTs evaluating interventions for shoulder disorders (6).

Secondary outcome measures

- Shoulder pain and disability at 6- and 12-months post-randomisation will be measured using the total SPADI score.

- Health-related quality of life at 12-weeks, 6- and 12- months post-randomisation will be measured using the EQ-5D-5L.

- Time to return to usual activities, including work and driving, will be measured via self-report questionnaire at 12-weeks, 6- and 12-months.

- Healthcare resource use at 12-weeks, 6- and 12-months will be measured via self-report questionnaire.

- Rotator cuff repair integrity (evidence of full-thickness re-tear; yes/ no) at 12-months will be assessed via diagnostic ultrasound scan.

- Number and nature of adverse events at 12-weeks, 6- and 12-months will be measured via self-report questionnaire and clinician report.

For peer review only

- Self-report time out of sling, measured in hours, over 4 weeks post-surgery via self-report diary.

Participants timeline

See Figures 1 and 2.

Please insert Figure 2 here

Figure 2. Trial schedule of assessments and outcomes.

For peer review only

For peer review only - http://bmjopen.bmj.com/site/about/guidelines.xhtml

Sample size

The sample size calculation was based on total SPADI score at 12 weeks, powered to detect a minimal clinically important difference of 8 points between groups (10). Assuming a standard deviation of 30 (the upper 80% confidence limit from our pilot study) (6) at 90% power and significance level 5%, and using an independent T-Test, results in 297 participants being needed per group (594 in total). However, using ANCOVA (primary analysis), adjusting for the baseline SPADI score, where correlation (r) between baseline and 12-weeks = 0.2 (data from pilot RaCeR RCT), the sample size was adjusted by (1-r2) plus one extra participant per group to 574 in total (11). In addition, adjusting for 10% non-response of SPADI questionnaire at 12 weeks, a target of 319 participants should be randomised per group, 638 in total.

Recruitment strategies

The Quintet Recruitment Intervention

We will implement the QRI aiming to optimise recruitment (8). Although our RaCeR pilot recruited 39% of those eligible, we anticipate challenges to recruitment in the main trial due to: (i) hesitance by surgeons to randomise patients (particularly older patients with larger rotator cuff tears), and (ii) challenges in participants accepting the randomised allocation due to perceived risks of individualised EPDR.

The QRI has been applied to over 25 RCTs to date, leading to insights about individual and generic recruitment issues and the development of targeted strategies to improve recruitment rates (12, 13). Rather than simply increasing the numbers of patients recruited, the QRI will aim to reduce ‘missed opportunities’ for enrolling eligible patients, while safeguarding informed consent. We will draw on insights from

For peer review only - http://bmjopen.bmj.com/site/about/guidelines.xhtml

For peer review only

previous application of QRI methods in RCTs, and the latest recruitment related evidence to develop materials and pre-emptive training which will support participant recruitment from the outset of RaCeR 2. Once sites open to recruitment, we will proceed to implement the QRI in two phases:

Phase 1: We will investigate recruitment issues that transpire 'in real time' throughout the remainder of the scheduled recruitment period. We will use mixed methods to investigate actual (rather than anticipated) issues hindering recruitment as the trial proceeds. Data collection will include:

• Semi-structured interviews with individuals involved in recruitment ('recruiters').

• Audio-recorded discussions between recruiters and potential participants about RaCeR 2.

• Mapping of recruitment pathways and screening log analysis.

Findings from these sources will be triangulated to generate an in-depth understanding of the 'root-causes' of key recruitment issues.

Phase 2: Using the results from phase 1, the QRI team will work closely with the TMG and Patient and Public Involvement (PPI) group to design and implement 'actions' to optimise recruitment. Actions may be applicable to all sites, specific sites, or individual recruiters, and will aim to increase the number of eligible patients approached, and/or improve conversion rates whilst safeguarding informed consent. The QRI phases will run iteratively. New avenues of enquiry will emerge throughout the conduct of the QRI, through discussion in feedback meetings and continued monitoring of screening logs.

For peer review only - http://bmjopen.bmj.com/site/about/guidelines.xhtml

We will pay close attention to screening log data before/after QRI actions to formatively evaluate the impact of actions, and the need for further investigation (Phase 1) or actions (Phase 2). Part of the QRI will entail up-front training for site staff as they open to recruitment. This training will evolve to become increasingly focused as we develop our understanding of recruitment issues, with a view to ensuring sites that open in the latter stages of the trial benefit from the QRI insights that have emerged to date.

Assignment of interventions: Allocation

Sequence generation

Upon completion of surgery, participants are randomised using minimisation. Participants are allocated on a 1:1 ratio, stratified by recruiting site and rotator cuff tear size; small (<1cm), medium (1cm to 3cm), large/massive (>3cm) or unknown.

Concealment mechanism

To ensure allocation concealment, randomisation is coordinated by Derby Clinical Trials Support Unit (DCTSU) remotely via an online randomisation system.

Implementation

The allocation sequence is generated by an online randomisation system. Following surgery, the local site team will explain to the participant their randomised allocation as well as other routine post-operative requirements. An exercise manual is provided

For peer review only - http://bmjopen.bmj.com/site/about/guidelines.xhtml

to all participants, along with the sling diary. Participants will complete the diary with the amount of time (hours and minutes) they were not wearing the sling at regular periods throughout the day.

Assignment of interventions: Blinding (masking)

RaCeR 2 is an open label RCT. No blinding of participants, clinicians, research team or oversight committees is in place.

Data collection, management, and analysis

Plans for assessment and collection of outcomes

Following consent, the baseline questionnaire will be completed prior to surgery in-person or remotely. Completion of the baseline questionnaire will require input from local site staff and participants. The questionnaire will include the SPADI and EQ-5D-5L validated questionnaires and demographic data. The SPADI has 13 items divided into two sub-scales: pain (5 items) and disability (8 items). The responses are indicated on a visual analogue scale (0 = no pain/no difficulty and 10 = worst imaginable pain/so difficult it requires help). The items are summed and converted to a total score out of 100, a high score indicates greater pain and disability (7). The EQ-5D-5L is a generic measure of health-related quality of life. It provides a single index value for health status that can be used in a clinical or health economic evaluation (14). The EQ-5D-5L consists of questions relating to five health domains (mobility, self-care, usual activities, pain/discomfort and anxiety/depression) and respondents rate their degree of impairment using five response levels (no problems,

For peer review only - http://bmjopen.bmj.com/site/about/guidelines.xhtml

slight, moderate, severe or extreme problems). The EQ-5D-5L is the National Institute for Health and Care Excellence's (NICE) preferred measure of health-related quality of life in adults.

Follow-up questionnaires, including SPADI, EQ-5D-5L, self-report questionnaire for healthcare resource use, time to return to usual activities (including work), and any adverse events, will be completed at 12 weeks, 6 and 12 months after randomisation (+4 weeks visit window to allow for reminders). This process will be coordinated centrally by the DCTSU. Follow-up questionnaires will be available in paper or electronic format. At 12 months following surgery participants will be asked to undergo an ultrasound scan.

Plans to promote participant retention and complete follow-up

If participants do not complete their questionnaires at the expected timepoints, they will be contacted at two weeks and a minimum data collection (SPADI and adverse events) will be attempted via telephone at three weeks.

Data management

A secure electronic software platform (Dacima™) will be used to store participant study data. Each participant is assigned a participant ID for use on study forms, other study documents and the electronic database.

For peer review only - http://bmjopen.bmj.com/site/about/guidelines.xhtml

Confidentiality

All documents will be stored safely in confidential conditions in accordance with the Data Protection Act 2018 and UK General Data Protection Regulation and retained according to national legislation.

Statistical methods

Primary and secondary outcome analysis

Primary analyses will be conducted according to the intention-to-treat analysis group. ANCOVA will be used to compare total SPADI scores between individualised EPDR versus standard rehabilitation at 12 weeks after randomisation, adjusting for baseline SPADI score.

Among other secondary analyses, time to return to usual activities (work & driving) will be analysed using Kaplan-Maier curves and log rank test. Logistic regression will be undertaken to test the association between treatment groups and re-tear at 12 months. Linear regression will be used to test the association between treatment groups and time out of sling over 4 weeks. Repeated measures ANCOVA will be used to test if any treatment effect exists and has been maintained up to 12 months in terms of SPADI, and EQ-5D-5L scores. ANCOVA will be used to compare total SPADI and EQ-5D-5L scores between the treatment groups at 6 and 12 months adjusting for baseline scores. Safety analysis will be undertaken based on the per protocol analysis group. Presence of Adverse events (AEs)/Serious adverse events (SAEs) and problems after surgery will be compared between the two groups at 12 weeks, 6- and 12-months using Chi-Squared test.

For peer review only - http://bmjopen.bmj.com/site/about/guidelines.xhtml

Interim analyses

Interim descriptive analysis will be undertaken at 6 months from the start of recruitment to assess the progression criteria of the internal pilot phase. This will not include any comparison of the patient reported outcomes between the randomised groups.

Methods for additional analyses

Exploratory subgroup analysis will be undertaken for the primary endpoint at 12 weeks including an interaction term in the ANCOVA model of "rotator cuff tear size" by "treatment group".

Definition of analysis population relating to protocol non-adherence and any statistical method to handle missing data

Per protocol analysis will consider patients with time out of sling of 222.6 hours or more over four weeks compared to those with time out of sling less than 222.6 hours base on the cut-off values from the RaCeR pilot (6). Missing values in the diary will not be included in the analysis.

Complete cases analysis will be undertaken as part of the primary endpoint analyses, where cases with missing values or those completed outside the four weeks window will be excluded in each analysis. If substantial missing data (>10% and <20%) are observed in SPADI at 12 weeks or a key prognostic covariate for the primary analysis, then multiple imputation using chained equations will be applied. Complete cases analysis will be undertaken for the secondary study outcomes.

For peer review only - http://bmjopen.bmj.com/site/about/guidelines.xhtml

Economic analysis

The perspective for both within-trial and model-based economic analyses will be that of the NHS and Personal Social Services (15). The economic analysis has three phases:

1) Development of a conceptual cost-effectiveness model structure: an initial conceptual cost-effectiveness model structure will be developed to estimate the long-term costs and quality-adjusted life year of EPDR and standard rehabilitation.

2) Within-trial cost-consequences analysis: health benefits will be quantified for changes in health-related quality of life, measured by the EQ-5D-5L. Healthcare resource use and costs observed during the trial period will be reported for each treatment group. Outcomes measured during the 12-month study period will be left undiscounted.

3) Model-based economic analysis: The long-term costs and health outcomes of EPDR and standard rehabilitation will be modelled for their impact on clinically relevant events (e.g., re-tear, re-operation), updating the state-transition model developed using the RaCeR pilot with parameters derived from data collected in RaCeR 2 and (where relevant) the published literature. Long-term predicted outcomes will be discounted at 3.5% per annum (15). The health economic analysis plan will be developed and finalised before analysis commenced and is anticipated to be disseminated in a separate publication.

For peer review only

Plans to give access to the full protocol, participant level data and statistical code

The full protocol is available at https://www.fundingawards.nihr.ac.uk/award/NIHR133874 . In the first instance, further requests for data can be made via the chief investigator (CL).

Oversight and monitoring

Composition of the coordinating centre and trial steering committee

The Chief Investigator (CL) is responsible for the conduct of the trial and will be supported by the TMG. The TMG oversees all day-to-day aspects of trial management and delivery. The independent TSC monitors the trial progress and ensures that is it is being conducted according to the protocol and the applicable regulations. The TSC has an independent chair (statistician), and four other independent members including a health economist, physiotherapist, surgeon and two PPI representatives as well as the Chief Investigator (non-independent). The TSC will meet annually. The Chief Investigator, Associate Investigator, Statistician and Trial Manager will attend the TSC meetings and report on trial progress.

Composition of the data monitoring committee, its role and reporting structure

Given the nature of RaCeR 2, a separate Data Monitoring Committee (DMC) will not be convened and the TSC will take on the data monitoring role, as agreed by the funder.

For peer review only - http://bmjopen.bmj.com/site/about/guidelines.xhtml

Adverse event reporting and harms

Number and nature of adverse events at 12-weeks, 6- and 12-months will be measured via self-report questionnaire and clinician report. Adverse events might include an increase in shoulder pain requiring additional care, e.g. prescribed medication or injection; infection up to 12-weeks post-surgery; other shoulder disorders, e.g. stiffness; rotator cuff re-rupture requiring additional care, e.g. injection, physiotherapy or surgery.

Frequency and plans for auditing trial conduct

Audits will be conducted by the Sponsor (University Hospitals of Derby & Burton NHS Foundation Trust) according to their audit plan; these may be central or site audits and may be trial or process-level audits.

Plans for communicating important protocol amendments to relevant parties

Substantial amendments will be submitted by the Sponsor to relevant regulatory bodies (Research Ethics Committee and Health Research Authority) for review and approval. The amendments will only be implemented after approval and a favourable opinion has been obtained. Non-substantial amendments will be submitted to the Health Research Authority for their approval/acknowledgment.

For peer review only

Ethics and dissemination

We were granted ethical approval by London-Stanmore Research Ethics Committee (23/LO/0195). We will disseminate findings from RaCeR 2 to stakeholders via peer-reviewed publications and presentations at national and international conferences. Our website (www.racer2study.co.uk) will serve as a hub to videos describing the trial results to support patient and clinical decision making.

For peer review only

Patient and public involvement

PPI was embedded throughout our RaCeR pilot (6). Our PPI group informed the choice of primary outcome, directed the timing of the intervention, the reporting of ultrasound scans, and the follow-up data collection methods. They will continue to be actively involved in all stages of RaCeR 2, including development of patient-facing documents and the qualitative interview schedule for the QRI. We will work collaboratively to co-create dissemination materials such as blogs and social media posts accessible to members of the public. The co-author MF is a TMG member. Our PPI group holds regular meetings, facilitated by our PPI lead (MM).

Discussion

RaCeR 2 will be the largest RCT in the world investigating rehabilitation after rotator cuff repair (5). The findings will inform national and international clinical practice. Our primary outcome assesses pain and disability. Our comprehensive dataset will assess other outcomes of interest to the clinical community, including rotator cuff

For peer review only - http://bmjopen.bmj.com/site/about/guidelines.xhtml

repair integrity, and the comparative cost effectiveness of individualised early patient directed rehabilitation versus standard rehabilitation.

Study Status

The RaCeR 2 trial (protocol version 2.2, 14th April 2023) opened to recruitment on the 1st June 2023 and is scheduled to remain open until 31st May 2025.

For peer review only

For peer review only - http://bmjopen.bmj.com/site/about/guidelines.xhtml

REFERENCES

1. Carr AJ, Cooper CD, Campbell MK, Rees JL, Moser J, Beard DJ, et al. Clinical effectiveness and cost-effectiveness of open and arthroscopic rotator cuff repair the UK Rotator Cuff Surgery (UKUFF) randomised trial . Health technology assessment (Winchester, England). 2015;19(80):1-218.
2. Karjalainen TV, Jain NB, Heikkinen J, Johnston RV, Page CM, Buchbinder R. Surgery for rotator cuff tears. The Cochrane database of systematic reviews. 2019;12(12):Cd013502.
3. HES. Hospital Episode Statistics. Hospital Admitted Patient Care Activity 2018-19 [Internet]. 2019 [cited 2020 May 18].
4. Littlewood C, Mazuquin B, Moffatt M, Bateman M. Rehabilitation following rotator cuff repair: A survey of current practice (2020). Musculoskeletal Care. 2021;19(2):165-71.
5. Mazuquin B, Moffatt M, Gill P, Selfe J, Rees J, Drew S, et al. Effectiveness of early versus delayed rehabilitation following rotator cuff repair: Systematic review and meta-analyses. PLOS ONE. 2021;16(5):e0252137.
6. Littlewood C, Bateman M, Butler-Walley S, Bathers S, Bromley K, Lewis M, et al. Rehabilitation following rotator cuff repair: A multi-centre pilot & feasibility randomised controlled trial (RaCeR). Clinical Rehabilitation. 2020:0269215520978859.
7. MacDermid JC, Solomon P, Prkachin K. The Shoulder Pain and Disability Index demonstrates factor, construct and longitudinal validity. BMC Musculoskelet Disord. 2006;7:12.
8. Donovan JL, Rooshenas L, Jepson M, Elliott D, Wade J, Avery K, et al. Optimising recruitment and informed consent in randomised controlled trials: the development and implementation of the Quintet Recruitment Intervention (QRI). Trials. 2016;17(1):283.
9. Chan AW, Tetzlaff JM, Altman DG, Laupacis A, Gøtzsche PC, Krleža-Jerić K, et al. SPIRIT 2013 statement: defining standard protocol items for clinical trials. Ann Intern Med. 2013;158(3):200-7.
10. Buchbinder R, Ramiro S, Huang H, Gagnier JJ, Jia Y, Whittle SL. Measures of Adult Shoulder Function. Arthritis Care & Research. 2020;72(S10):250-93.
11. Borm GF, Fransen J, Lemmens WA. A simple sample size formula for analysis of covariance in randomized clinical trials. J Clin Epidemiol. 2007;60(12):1234-8.
12. Rooshenas L, Scott LJ, Blazeby JM, Rogers CA, Tilling KM, Husbands S, et al. The QuinteT Recruitment Intervention supported five randomized trials to recruit to target: a mixed-methods evaluation. Journal of Clinical Epidemiology. 2019;106:108-20.
13. Rooshenas L, Elliott D, Wade J, Jepson M, Paramasivan S, Strong S, et al. Conveying Equipoise during Recruitment for Clinical Trials: Qualitative Synthesis of Clinicians' Practices across Six Randomised Controlled Trials. PLOS Medicine. 2016;13(10):e1002147.
14. Hernández Alava M, Wailoo AJ, Ara R. Tails from the peak district: adjusted limited dependent variable mixture models of EQ-5D questionnaire health state utility values. Value in health : the journal of the International Society for Pharmacoeconomics and Outcomes Research. 2012;15(3):550-61.

For peer review only - http://bmjopen.bmj.com/site/about/guidelines.xhtml

15. National Institute for Health and Clinical Excellence. Guide to the methods of technology appraisal, 2013.

For peer review only

Acknowledgements: NA

Authors' contributions: CL, MB, NF, BM, MM, AR, AM, VG, JR, ST and AF conceived of the study and were involved, alongside RS, KI and MF in developing the design and protocol. CL, BM, MM, AM, VG, AR, NF, JR, SD, MB and AF secured funding for the study. BM drafted the manuscript and all other authors reviewed and provided feedback on drafts. All authors read and approved the final version of the manuscript.

Funding statement: This trial is funded by the NIHR HTA (Ref: 133874). The views expressed are those of the author(s) and not necessarily those of the NIHR, the Department of Health and Social Care, or the NHS.

Availability of data and materials: Data produced from the trial will be made available to other researchers upon request, subject to approval by the Sponsor.

Ethics approval and consent to participate: Ethical approval was granted by London - Stanmore Research Ethics Committee (23/LO/0195). All participants must provide consent before participating, after receiving a full written and verbal explanation of the study's aims, procedures and risks.

Consent for publication: Consent for publication is not applicable as there are no identifying images or other personal details of participants presented.

Competing interests:

All authors declare support from the National Institute of Health and Care Research for the present manuscript.

AM is non-executive director of the ISPOR.

JR is Past President of the British Elbow and Shoulder Society (2021-2023).

For peer review only - http://bmjopen.bmj.com/site/about/guidelines.xhtml

SD reports education consultancy contracts with Stryker, Smith and Nephew and Arthrex for teaching and training. SD is the president of the British Elbow and Shoulder Society (2023-2025).

Word count: 4106 words

For peer review only

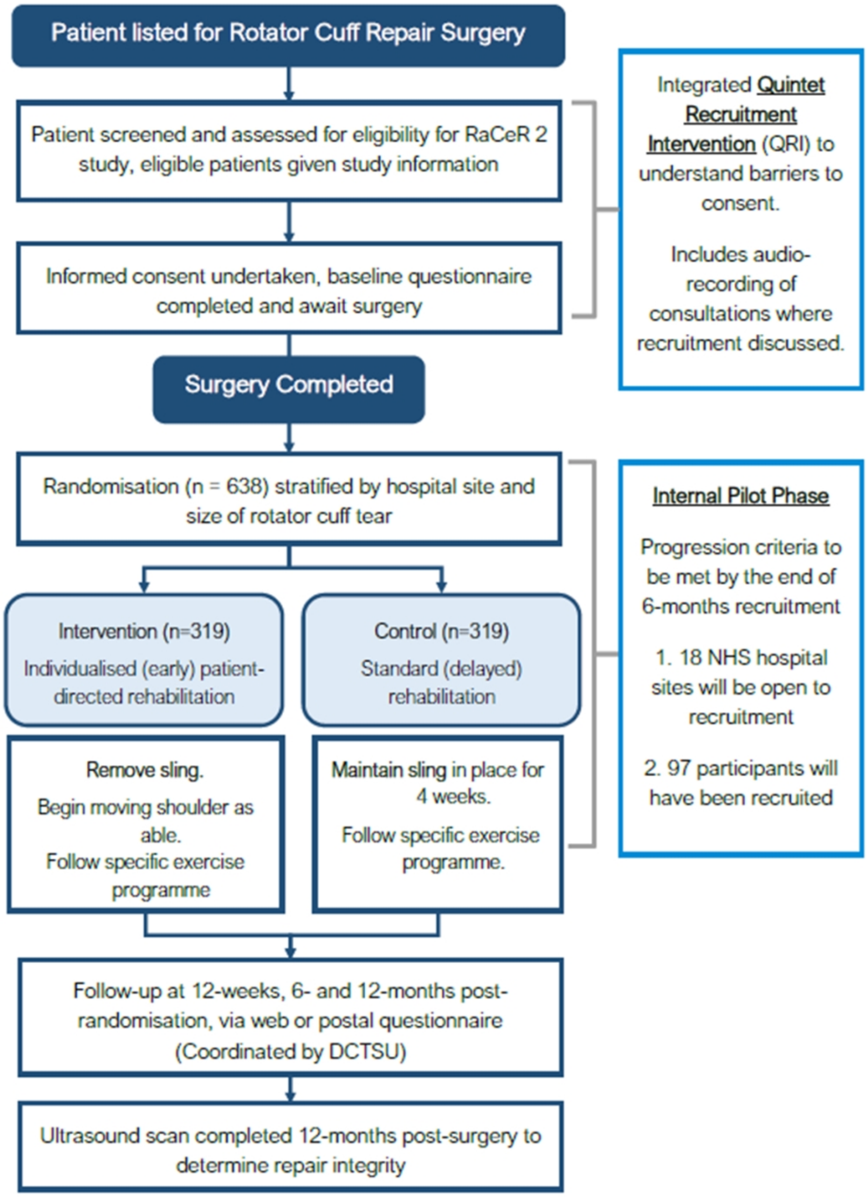

Figure 1. Study flow diagram.

100x135mm (300 x 300 DPI)

	Timepoint						
	Screening	Baseline	Surgery	4 weeks	12 weeks	6 months	12 months
**ENROLMENT**							
Eligibility	X						
Participant invitation	X						
Screening data collected	X						
Recording of recruitment appointment/ Informed consent (QRI)		X					
Randomisation			X				
**INTERVENTIONS**							
Early patient-directed rehabilitation			◆———	———	———◆		
Standard rehabilitation			◆———	———	———◆		
**ASSESSMENTS**							
Baseline questionnaire		X					
SPADI		X			X	X	X
EQ-5D-5L		X			X	X	X
Sling-use diary			◆———	———◆			
Adverse event questionnaire					X	X	X
Adverse event assessments (by clinicians)			◆———	———	———	———	———◆
Healthcare resources use					X	X	X
Ultrasound imaging							X
Assessment of treatment fidelity (by PIs)							X

PI: Principal Investigator, QRI: Quintet Recruitment Intervention, SPADI: Shoulder Pain and Disability Index, EQ-5D-5L: Euroqol five dimensions five levels.

Figure 2. Trial schedule of assessments and outcomes.

272x155mm (300 x 300 DPI)

Derby Clinical Trials Support Unit

University Hospitals of Derby and Burton NHS Foundation Trust

RaCeR 2 – Rehabilitation following Rotator Cuff Repair	
CONSENT FORM	
Local Researcher (PI):	
Participant ID:	

RaCeR 2 Rehabilitation following Rotator Cuff Repair

Please sign your initials in each box below to indicate your agreement:

- **I confirm** that I have read or have had read to me, the patient information leaflet (v2.1 05/Apr/2023) for the RaCeR 2 study, including information about the team recording our conversations. I have had the opportunity to think about the study, ask questions and have had these answered to my satisfaction. ☐

Consent for audio-recording study discussions (Quintet Recruitment Intervention – QRI)

The following section includes details of the audio recording of the conversations you have with the research team. ***Staff to tick here if this is not applicable:*** ☐

- **I consent** to the recording of discussions with me about the RaCeR 2 study and that these recordings will be written up by the study team or their authorised representatives. Yes ☐ No ☐

<u>If you say no, you can move to the next page and ignore the following two points.</u>

- **I understand** the recording of these discussions is voluntary and I am free to withdraw at any time, without giving any reason, without my medical care or legal rights being affected. ☐

- **I understand** that the recordings and write ups, may be used to support training, teaching or be shared with other researchers but I will not be identifiable from these. ☐

________________ ________________ ________________

Name of Participant Signature Date (DD/MMM/YYYY)

Consent taken in person ☐ *Remotely* ☐ *Consent taken verbally* ☐

I am confirming that if consent is taken remotely, I will ensure a signed copy is sent to the participant.

________________ ________________ ________________

When completed: 1 for participant; 1 for researcher site file; 1 to be kept in medical notes.

FUNDED BY NIHR National Institute for Health and Care Research

For peer review only - http://bmjopen.bmj.com/site/about/guidelines.xhtml

Derby Clinical Trials Support Unit

University Hospitals of Derby and Burton NHS Foundation Trust

Name of Person seeking consent | Signature | Date (DD/MMM/YYYY)

Consent for RaCeR 2 study participation

<u>If you do not wish to take part, you can ignore this page.</u>

- **I understand** that my participation in the RaCeR 2 study is voluntary and that I am free to withdraw at any time without giving any reason, without my medical care or legal rights being affected. ☐
- **I understand** that relevant sections of my medical notes and data collected, may be looked at by authorised members of the research team from regulatory authorities or from the NHS Trust, where it is relevant to my taking part in this research. I give permission for these individuals to have access to my records, which includes identifiable information about me. ☐
- **I understand** that the information held and maintained by Derby Clinical Trials Support Unit may be used to contact me about completing the questionnaires required for the study. ☐
- **I agree** to my GP being informed of my participation in the RaCeR 2 study. ☐
- **I would like to** receive updates throughout the study, including the results Yes ☐ No ☐
- **I consent** to take part in the RaCeR 2 Study ☐

Name of Participant | Signature | Date (DD/MMM/YYYY)

Consent taken in person ☐ *remotely* ☐

Consent taken verbally ☐

I am confirming that if consent is taken remotely, I will ensure a signed copy is sent to the participant.

When completed: 1 for participant; 1 for researcher site file; 1 to be kept in medical notes.

RaCeR 2 Consent Form v2.1 05/APR/2023
(IRAS ID: 318438)

FUNDED BY NIHR | National Institute for Health and Care Research

For peer review only - http://bmjopen.bmj.com/site/about/guidelines.xhtml

For peer review only

Derby Clinical Trials Support Unit

University Hospitals of Derby and Burton NHS Foundation Trust

Name of Person seeking consent	Signature	Date (DD/MMM/YYYY)

For peer review only

When completed: 1 for participant; 1 for researcher site file; 1 to be kept in medical notes.

FUNDED BY NIHR | National Institute for Health and Care Research

For peer review only - http://bmjopen.bmj.com/site/about/guidelines.xhtml

STANDARD PROTOCOL ITEMS: RECOMMENDATIONS FOR INTERVENTIONAL TRIALS

SPIRIT 2013 Checklist: Recommended items to address in a clinical trial protocol and related documents*

Section/item	ItemNo	Description	Page
**Administrative information**			
Title	1	Descriptive title identifying the study design, population, interventions, and, if applicable, trial acronym	1
Trial registration	2a	Trial identifier and registry name. If not yet registered, name of intended registry	2
	2b	All items from the World Health Organization Trial Registration Data Set	NA
Protocol version	3	Date and version identifier	25
Funding	4	Sources and types of financial, material, and other support	28
Roles and responsibilities	5a	Names, affiliations, and roles of protocol contributors	1 and 28
	5b	Name and contact information for the trial sponsor	23
	5c	Role of study sponsor and funders, if any, in study design; collection, management, analysis, and interpretation of data; writing of the report; and the decision to submit the report for publication, including whether they will have ultimate authority over any of these activities	28
	5d	Composition, roles, and responsibilities of the coordinating centre, steering committee, endpoint adjudication committee, data management team, and other individuals or groups overseeing the trial, if applicable (see Item 21a for data monitoring committee)	22
**Introduction**			
Background and rationale	6a	Description of research question and justification for undertaking the trial, including summary of relevant studies (published and unpublished) examining benefits and harms for each intervention	3-4
	6b	Explanation for choice of comparators	9-10

For peer review only

Objectives	7	Specific objectives or hypotheses	4
Trial design	8	Description of trial design including type of trial (eg, parallel group, crossover, factorial, single group), allocation ratio, and framework (eg, superiority, equivalence, noninferiority, exploratory)	4
**Methods: Participants, interventions, and outcomes**			
Study setting	9	Description of study settings (eg, community clinic, academic hospital) and list of countries where data will be collected. Reference to where list of study sites can be obtained	5
Eligibility criteria	10	Inclusion and exclusion criteria for participants. If applicable, eligibility criteria for study centres and individuals who will perform the interventions (eg, surgeons, psychotherapists)	6
Interventions	11a	Interventions for each group with sufficient detail to allow replication, including how and when they will be administered	9
	11b	Criteria for discontinuing or modifying allocated interventions for a given trial participant (eg, drug dose change in response to harms, participant request, or improving/worsening disease)	11
	11c	Strategies to improve adherence to intervention protocols, and any procedures for monitoring adherence (eg, drug tablet return, laboratory tests)	11
	11d	Relevant concomitant care and interventions that are permitted or prohibited during the trial	11
Outcomes	12	Primary, secondary, and other outcomes, including the specific measurement variable (eg, systolic blood pressure), analysis metric (eg, change from baseline, final value, time to event), method of aggregation (eg, median, proportion), and time point for each outcome. Explanation of the clinical relevance of chosen efficacy and harm outcomes is strongly recommended	12
Participant timeline	13	Time schedule of enrolment, interventions (including any run-ins and washouts), assessments, and visits for participants. A schematic diagram is highly recommended (see Figure)	12-13 (Figure 2)

Sample size	14	Estimated number of participants needed to achieve study objectives and how it was determined, including clinical and statistical assumptions supporting any sample size calculations	14
Recruitment	15	Strategies for achieving adequate participant enrolment to reach target sample size	14-16

Methods: Assignment of interventions (for controlled trials)

Allocation:

Sequence generation	16a	Method of generating the allocation sequence (eg, computer-generated random numbers), and list of any factors for stratification. To reduce predictability of a random sequence, details of any planned restriction (eg, blocking) should be provided in a separate document that is unavailable to those who enrol participants or assign interventions	16
Allocation concealment mechanism	16b	Mechanism of implementing the allocation sequence (eg, central telephone; sequentially numbered, opaque, sealed envelopes), describing any steps to conceal the sequence until interventions are assigned	16
Implementation	16c	Who will generate the allocation sequence, who will enrol participants, and who will assign participants to interventions	16
Blinding (masking)	17a	Who will be blinded after assignment to interventions (eg, trial participants, care providers, outcome assessors, data analysts), and how	17
	17b	If blinded, circumstances under which unblinding is permissible, and procedure for revealing a participant’s allocated intervention during the trial	NA

Methods: Data collection, management, and analysis

Data collection methods	18a	Plans for assessment and collection of outcome, baseline, and other trial data, including any related processes to promote data quality (eg, duplicate measurements, training of assessors) and a description of study instruments (eg, questionnaires, laboratory tests) along with their reliability and validity, if known. Reference to where data collection forms can be found, if not in the protocol	17
	18b	Plans to promote participant retention and complete follow-up, including list of any outcome data to be collected for participants who discontinue or deviate from intervention protocols	18

For peer review only - http://bmjopen.bmj.com/site/about/guidelines.xhtml

Data management	19	Plans for data entry, coding, security, and storage, including any related processes to promote data quality (eg, double data entry; range checks for data values). Reference to where details of data management procedures can be found, if not in the protocol	18
Statistical methods	20a	Statistical methods for analysing primary and secondary outcomes. Reference to where other details of the statistical analysis plan can be found, if not in the protocol	19
	20b	Methods for any additional analyses (eg, subgroup and adjusted analyses)	20
	20c	Definition of analysis population relating to protocol non-adherence (eg, as randomised analysis), and any statistical methods to handle missing data (eg, multiple imputation)	20
**Methods: Monitoring**			
Data monitoring	21a	Composition of data monitoring committee (DMC); summary of its role and reporting structure; statement of whether it is independent from the sponsor and competing interests; and reference to where further details about its charter can be found, if not in the protocol. Alternatively, an explanation of why a DMC is not needed	22
	21b	Description of any interim analyses and stopping guidelines, including who will have access to these interim results and make the final decision to terminate the trial	20
Harms	22	Plans for collecting, assessing, reporting, and managing solicited and spontaneously reported adverse events and other unintended effects of trial interventions or trial conduct	23
Auditing	23	Frequency and procedures for auditing trial conduct, if any, and whether the process will be independent from investigators and the sponsor	23
**Ethics and dissemination**			
Research ethics approval	24	Plans for seeking research ethics committee/institutional review board (REC/IRB) approval	28

Protocol amendments	25	Plans for communicating important protocol modifications (eg, changes to eligibility criteria, outcomes, analyses) to relevant parties (eg, investigators, REC/IRBs, trial participants, trial registries, journals, regulators)	23
Consent or assent	26a	Who will obtain informed consent or assent from potential trial participants or authorised surrogates, and how (see Item 32)	6
	26b	Additional consent provisions for collection and use of participant data and biological specimens in ancillary studies, if applicable	NA
Confidentiality	27	How personal information about potential and enrolled participants will be collected, shared, and maintained in order to protect confidentiality before, during, and after the trial	18-19
Declaration of interests	28	Financial and other competing interests for principal investigators for the overall trial and each study site	28
Access to data	29	Statement of who will have access to the final trial dataset, and disclosure of contractual agreements that limit such access for investigators	22
Ancillary and post-trial care	30	Provisions, if any, for ancillary and post-trial care, and for compensation to those who suffer harm from trial participation	11
Dissemination policy	31a	Plans for investigators and sponsor to communicate trial results to participants, healthcare professionals, the public, and other relevant groups (eg, via publication, reporting in results databases, or other data sharing arrangements), including any publication restrictions	24
	31b	Authorship eligibility guidelines and any intended use of professional writers	28
	31c	Plans, if any, for granting public access to the full protocol, participant-level dataset, and statistical code	22
**Appendices**			
Informed consent materials	32	Model consent form and other related documentation given to participants and authorised surrogates	Not available
Biological specimens	33	Plans for collection, laboratory evaluation, and storage of biological specimens for genetic or molecular analysis in the current trial and for future use in ancillary studies, if applicable	NA

For peer review only

*It is strongly recommended that this checklist be read in conjunction with the SPIRIT 2013 Explanation & Elaboration for important clarification on the items. Amendments to the protocol should be tracked and dated. The SPIRIT checklist is copyrighted by the SPIRIT Group under the Creative Commons "Attribution-NonCommercial-NoDerivs 3.0 Unported" license.

For peer review only
